# Time-Restricted Eating Combined with Exercise Reduces Menopausal Symptoms and Improves Quality of Life More than Exercise Alone in Menopausal Women: A Quasi-Randomized Controlled Trial

**DOI:** 10.3390/nu17203274

**Published:** 2025-10-18

**Authors:** Beata Jóźwiak, Adam Szulc, Ida Laudańska-Krzemińska

**Affiliations:** 1 Department of Physical Activity and Health Promotion Science, Poznan University of Physical Education, 61-871 Poznań, Poland; idakrzeminska@awf.poznan.pl; 2 Warsaw School of Economics, Institute of Statistics and Demography, 02-554 Warsaw, Poland; aszulc@sgh.waw.pl

**Keywords:** diet, physical activity, menopause, climacteric symptoms, intermittent fasting

## Abstract

**Background**: Menopause is often accompanied by menopausal symptoms and reduced quality of life. Studies on the combined effects of time-restricted eating and exercise in this population are lacking. This approach may provide additive preventive benefits by aligning nutritional timing with exercise to improve health and well-being in menopausal women. We aimed to assess whether a combined intervention is more effective than exercise alone in reducing menopausal symptoms and improving quality of life. **Methods**: This study examined the effects of a time-restricted eating protocol (16:8) combined with a resistance and endurance circuit training program in menopausal women. Symptoms were assessed using the Menopause Rating Scale (MRS), and quality of life was evaluated with the Menopause-Specific Quality of Life Questionnaire (MENQOL). Participants (*n* = 54) were quasi-randomly assigned to a combination group (exercise + time-restricted eating; *n* = 24) or an exercise group (exercise only; *n* = 30), with allocation influenced by participant preference. **Results**: The reduction in the total MRS score, as well as in the psychological and somatic MRS subdomains, was significantly greater in the combination group than in the exercise group (*p* = 0.008, *p* = 0.009, *p* = 0.007, respectively). No significant difference was observed in the urogenital domain. For MENQOL, post-intervention scores in the physical and psychosocial subdomains were significantly lower in the combination group compared with the exercise group (*p* = 0.013, *p* = 0.002, respectively), while no significant differences were found in the vasomotor and sexual subdomains. **Conclusions**: These findings suggest that integrating time-restricted eating with exercise results in greater alleviation of menopausal symptoms and improvements in quality of life compared to exercise alone in menopausal women.

## 1. Introduction

Menopause signifies changes in a woman’s reproductive system that have a far-reaching effect on physical, psychological, and social health [1]. Menopause is a critical life event in aging women and has been suggested to be a period of declining wellbeing and quality of life [2]. Many women experience pronounced menopausal symptoms, which cause them to seek medical care [3]. Treatment options for menopausal symptoms range from hormone replacement therapy to non-hormonal alternatives and lifestyle modifications [4]. An example of such a lifestyle modification is physical exercise which has been suggested to be an effective strategy for attenuating climacteric symptoms, decreasing sleep disturbances, and improving quality of life and life satisfaction in menopausal women [5,6,7,8]. Another example is dietary patterns that may support health of menopausal women and reduce the severity of menopausal symptoms [9,10,11]. Recently, combined therapies and lifestyle modifications have garnered increased attention, as integrating multiple methods can maximize synergistic effects of each intervention [12,13]. This approach merits further investigation and application.

Intermittent fasting (IF) is an eating pattern that consists of periods of consumption and periods of fasting [14]. Time-restricted eating (TRE) is a type of intermittent fasting and involves consuming meals within a limited time frame (eating window), followed by a period of fasting. Depending on the chosen model, the eating window ranges from 6 to 12 h, with an average of 8 h [15]. Recently, IF has gained significant attention from researchers due to its promising effects on health and metabolism [16]. IF has shown positive effects particularly on cardiometabolic health [14,17]. Despite these positive health outcomes, its effect on menopausal symptoms and quality of life among menopausal women has not been investigated before.

Autophagy is a cellular degradation and recycling process that plays a key role not only in the elimination of pathogens (xenophagy) but also in various other defensive mechanisms of the organism. It is involved in the regulation of inflammatory responses and interacts with other innate immunity pathways [18]. Additionally, autophagy influences the regulation of adaptive immunity and is essential for the proper development and function of the immune system, particularly under conditions of cellular stress such as starvation [19].

Autophagy exerts anti-inflammatory effects by inhibiting the activation of inflammasomes—structures responsible for initiating inflammatory responses—and regulating type I interferon responses, which are crucial for antiviral defense [20]. This process attenuates inflammation, a mechanism particularly relevant in the context of aging and the presence of chronic inflammatory conditions [21].

During fasting episodes, the limited availability of macro- and micronutrients triggers a range of adaptive cellular mechanisms. One of the key responses is the activation of adenosine monophosphate-activated protein kinase (AMPK), which senses low energy levels and inhibits the mechanistic target of rapamycin (mTOR) pathway—the primary nutrient sensor within the cell [22]. Inhibition of mTOR leads to the induction of autophagy through activation of the Unc-51-like kinase 1 (ULK1) complex, which initiates the process [23]. Under nutrient-rich conditions, mTOR phosphorylates ULK1, thereby suppressing autophagy, whereas during fasting, mTOR deactivation combined with concurrent AMPK activation results in ULK1 activation and autophagy initiation [23]. This process not only facilitates the removal of damaged cellular components but also reduces chronic inflammation and, in the long term, improves metabolic health and extends lifespan [22].

Intermittent fasting (IF) represents a powerful natural inducer of autophagy [24]. Throughout periods of IF, the body transitions its metabolic processes to favor fat utilization, resulting in the generation of ketone bodies like beta-hydroxybutyrate (BHB), which possesses additional anti-inflammatory effects [23]. Similarly, calorie restriction (CR) promotes autophagy, improves antigen presentation to CD4+ T cells [25], decreases circulating monocyte counts, and lowers their metabolic and inflammatory responses in both humans and mice [26].

Evidence suggests that women have inherently lower levels of basal autophagy compared to men, with these differences emerging early in life [22]. Estrogens play an important role in the regulation of autophagy by promoting the maturation of autophagosomes and enhancing lysosomal efficiency in the clearance of pathological proteins. Following menopause, the decline in estrogen leads to impaired mitochondrial autophagy, increased beta-amyloid production, and reduced cellular clearance of damaged structures, thereby accelerating the aging process [2,5]. The loss of estrogens also contributes to increased insulin resistance, which in turn excessively activates the mTOR signaling pathway, further inhibiting autophagy. Sex hormones regulate stress-induced autophagy through two key signaling pathways: phosphoinositide 3-kinase (PI3K), which governs cell growth and metabolism, and AMPK, which senses energy deficits and activates autophagy [27].

Mounting evidence suggests that certain organ systems respond similarly to intermittent fasting and aerobic exercise. Both induce beneficial cellular changes, including inhibition of the mTOR pathway, stimulation of autophagy, and promotion of mitochondrial biogenesis [28].

Previous studies on the effects of combined diet-exercise interventions on menopausal symptoms provided evidence that multidomain interventions are beneficial for managing menopausal symptoms. However, further studies are needed to evaluate synergistic potential of lifestyle modifications [29]. Research suggests that IF may enhance beneficial effects of physical activity on health [13]. Combining IF with exercise has been shown to facilitate fat mass reduction while preserving lean body mass [30,31]. There is a need to investigate whether this combined approach can effectively alleviate menopausal symptoms, support overall health, and enhance quality of life during a sensitive period of menopausal transition.

Although the beneficial effects of physical activity and intermittent fasting on overall health in humans are well-known [32], it remains unclear whether TRE combined with exercise can modify or augment menopausal symptoms and quality of life in menopausal women compared with exercise alone. Considering this, the present study aimed to determine whether implementing both exercise and TRE, rather than exercise alone, produces a significant effect on menopausal symptoms and quality of life in women experiencing menopause. It was hypothesized that the combined intervention would result in greater improvements in menopausal symptoms and quality of life compared to exercise alone.

## 2. Materials and Methods

### 2.1. Study Design and Procedures

A 12-week quasi-randomized controlled parallel between-group trial was conducted to test the effect of exercise (exercise group), and TRE combined with exercise (combination group) on menopausal symptoms and quality of life in menopausal women. Eligible participants were distributed into an exercise group or a combination group. The combination group participated in both TRE and exercise interventions, whereas the exercise group engaged in the exercise intervention only. Collection of the data, exercise intervention and study procedures took place at Poznan University of Physical Education. The trial was run in three bouts. The first occurred from October to December 2021, the second from January to March 2022, and the third from October to December 2022. Recruitment for each bout took place during the four weeks preceding its start, using advertisements on the university website. Participants were assigned to groups using an alternating sequence, with final allocation partly guided by their personal preferences to improve adherence and reduce dropout. This approach was considered important for ethical and practical reasons. The sequence was implemented by the study coordinator to ensure transparency and consistency. To address high dropout rates, additional participants were assigned to groups during the second and third recruitment waves, maintaining a near 1:1 allocation ratio and ensuring comparable group sizes at the end of the study. Most scientific workers were blinded to allocation into the exercise and combination group, except for participants and staff supervising the training sessions. Outcome assessors were blinded to group allocation.

### 2.2. Primary and Secondary Outcome Measures

The primary outcome measures were the change from baseline to week 12 in Menopause Rating Scale (MRS) and Menopause-Specific Quality of Life (MENQOL).

The secondary outcome measures were Satisfaction with Life Scale (SWLS), Fatigue Assessment Scale (FAS), General Health Questionnaire (GHQ-12), Single-Item Sleep Quality Scale (SQS), lymphocyte-to-monocyte ratio (LMR), and body mass index (BMI).

### 2.3. Participants

**Figure 1:**
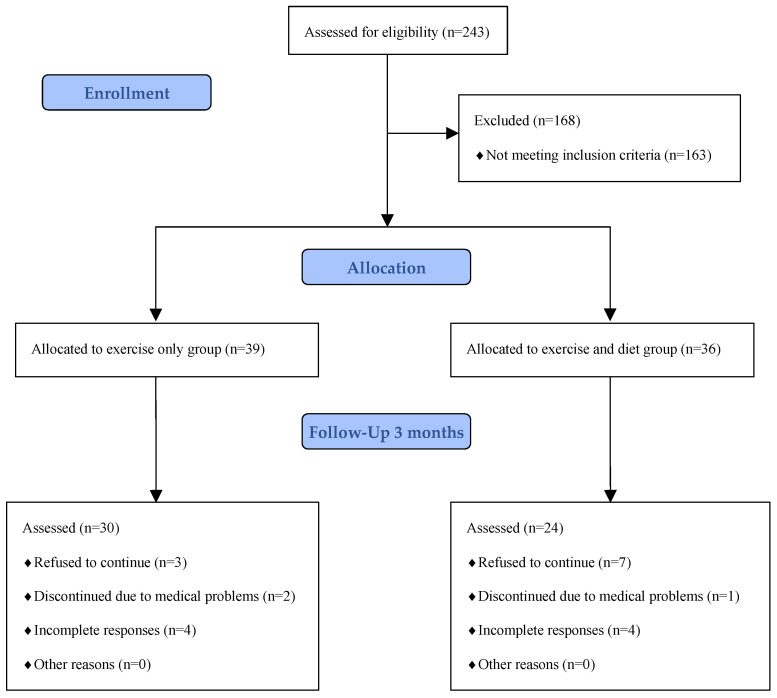
Flow diagram of the study following CONSORT guidelines.

Sample size calculations (G*Power 3.1.7) revealed that a minimum sample size of 50 participants would be appropriate to detect significant differences between the groups, assuming an effect size (ES) η_p_^2^ = 0.15, a type I error of 0.05, and a test power of 0.80. On the basis of a prediction of a dropout rate of 20%, an estimated 63 participants were required. Recruitment took place in Poznan, Poland, through advertisements on the university website. Eligible participants were women aged 41–61 years classified as perimenopausal (irregular menstrual bleeding within the past 12 months), menopausal (no menstrual bleeding for 12 consecutive months), or postmenopausal (no menstrual bleeding for more than one year), and inactive or lightly active. Global Physical Activity Questionnaire (GPAQ) [33] was used to assess level of physical activity. Exclusion criteria included night-shift work, eating disorders, ≥4 kg body weight change within 3 months, use of lipid/glucose lowering or antihypertensive medication, diabetes, cardiovascular disease, hormonal contraception, hormone replacement therapy. Women contraindicated for exercise or fasting were excluded after physician evaluation. Of 243 individuals, 80 met all criteria (Figure 1). The study was approved by Bioethical Committee of the Poznan University of Medical Sciences (KB-179/21) in accordance with Declaration of Helsinki [34], and all participants provided written informed consent. The trial was registered on ClinicalTrials.gov (TRN: NCT06138015).

### 2.4. Questionnaire Assessment

The questionnaire assessments were performed before the start of the intervention and at the end of 12 weeks.

Menopausal symptoms were evaluated using the Menopause Rating Scale (MRS) [35], a questionnaire comprising 11 items divided into three categories: somatic, psychological, and urogenital. Each item represents a menopausal symptom rated on a 5-point Likert scale ranging from 0 (no symptom) to 4 (severe). The somatic category includes four questions addressing hot flashes and sweating, heart issues, sleep disturbances, and joint and muscle problems. The psychological category has four questions, focusing on depressive symptoms, irritability, anxiety and physical or mental exhaustion. The urogenital category includes three questions related to sexual issues, bladder issues, and vaginal dryness. Higher total and subscale scores indicate greater severity of menopausal symptoms.

The Menopause-Specific Quality of Life (MENQOL) [36] questionnaire is designed to assess the impact of menopausal symptoms on quality of life. It comprises 29 items, each rated on a 7-point scale from 0 (not bothered at all) to 6 (bothered a lot). The questionnaire covers four domains: vasomotor (3 items: hot flushes, night sweats, sweating), psychosocial (6 items: anxiety, poor memory, accomplishing less than in the past, feeling depressed, being impatient, feelings of wanting to be alone), physical (16 items: flatulence, aching in muscles and joints, feeling tired, difficulty sleeping, aches in back neck or head, decrease in strength or stamina, feeling a lack of energy, dry skin, weight gain, facial hair, changes in appearance of skin, feeling bloated, low backache, frequent urination, involuntary urination), and sexual (3 items: change in sexual desire, vaginal dryness, avoiding intimacy), each addressing specific symptoms that may be experienced during menopause. Higher scores indicate a greater negative impact of menopausal symptoms on quality of life.

The Satisfaction with Life Scale (SWLS) [37] is a 5-item instrument measuring overall life satisfaction based on individual standards and expectations, using a 5-point Likert scale, with responses ranging from 1 (never true) to 7 (always true). Higher scores indicate greater life satisfaction.

Fatigue Assessment Scale (FAS) [38] is a tool used to assess chronic fatigue symptoms. It consists of 10 items, each with 5 response options ranging from 1 (never) to 5 (always). The FAS provides scores for two subscales: physical fatigue and mental fatigue, with each subscale containing 5 items. Higher scores indicate a higher level of fatigue.

General Health Questionnaire (GHQ-12) [39] is a tool used to assess psychological well-being. It comprises 12 items scored on a Likert scale from 0 to 3. The scale includes 6 positively worded and 6 negatively worded questions. Higher scores indicate lower levels of psychological well-being.

Single-Item Sleep Quality Scale (SQS) [40] is a questionnaire that utilizes a visual analog scale (VAS) with discrete intervals. Respondents are instructed to assess their overall sleep quality over the past 7 days using this scale, marking an integer score from 0 (terrible) to 10 (excellent). When evaluating their sleep quality, respondents are instructed to consider key factors such as total number of sleep hours, ease of falling asleep, frequency of nighttime awakenings (excluding bathroom visits), instances of waking up earlier than needed, and how refreshing their sleep was.

### 2.5. Blood Collection and BMI Measurement

Blood samples were collected by an experienced nurse before and after the intervention following an overnight fast (≥10 h). Five mL of blood were drawn into EDTA tubes and analyzed for lymphocyte and monocyte counts using an enzymatic system and absorbance spectrophotometry. The LMR was calculated using the formula: [LMR = absolute lymphocyte count/absolute monocyte count]. Height was measured using a stadiometer, and body mass was measured with a scale. BMI was calculated using the formula: [BMI = weight [kg]/height [m]^2^].

### 2.6. Diet Protocol

Only the combination group took part in the TRE intervention, which involved eating within an 8-h window or less and fasting for the remainder of the day. Participants began their eating window at a self-selected time. The type and amount of food were also self-selected. Participants were instructed not to alter the types of foods typically consumed. Nutritional data from participants was gathered weekly through an online survey, where they recorded the times of their first and last meals for each day. Exercise group subjects were asked to maintain their regular eating habits. Before and after the experiment, the participants of both groups completed the Questionnaire for Dietary Habits, Lifestyle, and Nutrition Knowledge Assessment (KomPAN) [41]. This questionnaire evaluates food frequency consumption (FFC), which provides information about the frequency of consumption of the 33 different food items. According to the KomPAN authors’ instruction, 10 of the 33 items were used to calculate the Pro-Healthy Index and fourteen of these items were used to calculate the Non-Healthy Index.

### 2.7. Exercise Protocol

Participants in the exercise group and in the combination group engaged in a supervised, moderate-intensity strength and endurance circuit training program twice weekly for 12 weeks. The training was conducted using eight machines that were a part of the MILON^®^ system in the following circuited sequence: cycle ergometer, abdominal crunch, leg curl, latissimus pulldown, elliptical machine, leg press, back extension, and leg abductor. Aerobic exercises were performed on the cycle ergometer and the elliptical machine. Strength exercises were performed on strength training machines and involved abdominal muscles, hamstrings, latissimus dorsi, quadriceps, erector spinae, gluteal muscles. Each exercise session included three circuited sequences, with specific durations: 1 min for strength exercises, 4 min for endurance exercises, and 30-s breaks after each exercise. Training intensity was individualized based on each participant’s age-predicted heart rate maximum (HRmax), calculated using Fox’s equation (Fox-HR_max_ = 220 − age). A Polar Heart Rate Monitor (Polar USA, Inc., Bethpage, NY, USA) was used to monitor each participant’s heart rate (HR) during sessions, and exercise intensity was adjusted accordingly. According to the American College of Sports Medicine (ACSM), moderate-intensity aerobic exercise is achieved when heart rate reaches 64–76% of age-predicted maximal heart rate. The intensity of the resistance training was individualized based on the muscle strength level of each participant, assessed by the one-repetition maximum (1RM). The resistance load was progressively increased every 4 weeks. The resistance training load was 50% of the 1RM during weeks 1–4, 60% of the 1RM during weeks 5–8, and 70% of the 1RM during weeks 9–12. Each exercise session lasted 55 min. Participants were also asked to maintain their regular activity habits. The participants were required to attend a minimum of 90% of the training sessions (at least 22 of 24 training sessions), with any participant not meeting this attendance threshold being excluded from the final analysis.

### 2.8. Statistical Analysis

Since assumption of normality was generally rejected in Shapiro–Wilk test, Mann–Whitney U test was applied in intragroup and between group comparisons. Chi-square contingency test was used to compare the prevalence of menopausal status among each group. The results are expressed as the means ±standard deviations and medians with upper and lower quartiles. For the Wilcoxon test, the effect size was calculated according to a formula r = |z|/sqrt(N) and interpreted as follows: trivial: r < 0.20, small: 0.20 ≤ r < 0.50, moderate: 0.50 ≤ r < 0.80 or large: r ≥ 0.80 [42,43]. The effect of TRE combined with exercise was examined using regression models that account for the influence of additional variables. In all models, the dependent variables were defined as the differences between baseline and final values of questionnaire assessment outcome measures. The key explanatory variable was a dummy indicating participation in the group combining exercise with TRE. Control variables included age, initial BMI, change in LMR, and an interaction term between change in LMR and TRE. These variables were included to account for potential confounding factors and explanatory mechanisms: age and BMI can influence the response to interventions, while change in LMR may help explain whether observed effects are associated with changes in systemic inflammation. The interaction term was included to test whether the effect of TRE depends on concurrent changes in inflammatory status. Unlike ANOVA, regression models estimate not only the presence but also strength and direction of relationships between variables. Moreover, regression is more flexible than ANOVA/ANCOVA regarding assumptions about functional form, normality of residuals, and homoscedasticity. These assumptions were tested using the RESET test (for the model specification), the Shapiro–Wilk test (for normality), and the Breusch-Pagan test with Koenker correction (for heteroscedasticity, particularly under non-normal residuals). No violations of the homoscedasticity assumption were found for any dependent variable. However, for two outcomes, the linear model required modification to a quadratic form. Specifically, a squared age term was added for the MENQOL sexual domain and a squared change in LMR was added for the MENQOL psychosocial domain. The magnitude of the analyzed effects can be assessed using the coefficient of determination (R-squared), which represents the proportion of variation in the dependent variable explained by variation in the independent variables. Broadly speaking, R-squared is analogous to eta-squared (SSB/SST) used in analysis of variance. Differences with a *p* value ≤ 0.05 were considered statistically significant. The calculations were performed using STATISTICA 13.3 (StatSoft, Inc., Tulsa, OK, USA) and StataNow 19.5 (StataCorp LLC, College Station, TX, USA).

## 3. Results

### 3.1. Participants

A total of 80 participants were recruited for this study, with an overall dropout rate of 33% by the end of 12-week period (Figure 1). The high dropout rate was attributed to participants’ personal circumstances and concerns related to the COVID-19 pandemic. Ultimately, 54 participants completed the study (Figure 1). Participants were monitored for unintended effects through twice-weekly check-ins with researchers during exercise sessions, and no serious unintended effects were reported. The average age of participants was 51.07 ± 4.66 years. Statistical analysis revealed that participants in the combination group were significantly older than in the exercise group (*p* = 0.031). The average BMI was 26.82 ± 5.45, with no significant difference in BMI averages between the groups at baseline (*p* = 0.247). No other significant differences between the combination group and the exercise group were identified at baseline, except for the score in the MENQOL sexual domain, with the average score in the combination group being significantly lower than in the exercise group (*p* = 0.013). In terms of menopausal status, the exercise group consisted of 40% perimenopausal/menopausal women and 60% postmenopausal women, whereas the combination group included 29% and 71%, respectively. Baseline distribution of menopausal status did not differ between groups (*p* = 0.407). Within the combination group, no baseline differences were observed between menopausal subgroups. In the exercise group, perimenopausal/menopausal participants had significantly lower total MRS score, MRS somatic, and urogenital domain scores than postmenopausal participants at baseline (*p* = 0.020, *p* = 0.046, *p* = 0.045, respectively).

### 3.2. Adherence to Exercise and/or Dietary Intervention

A total of 24 supervised training sessions were conducted, with participants allowed to miss up to 2 sessions. Exercise compliance was assessed by recording attendance at each session, and both groups completed over 95% of the sessions. If an exercise session was missed, the participant was required to compensate for the missed session. Almost 80% of participants exercised at moderate or higher intensities for at least 50% of the time during the sessions, with no difference in exercise intensity between the groups (*p* = 0.537). Adherence to the dietary regimen was lower than to exercise. Participants in the combination group followed the prescribed 8-h eating window on over 62% of days and the 9-h eating window on over 86% of days. Before the intervention, the mean eating window was 12 h 25 min. The KomPAN food frequency questionnaire was used to assess baseline and post-intervention intensities for pro-healthy and non-healthy food consumption. No significant changes in food frequency patterns categorized as pro-healthy (*p* = 0.141) or non-healthy (*p* = 0.072) were observed in the combination group, suggesting that the quality and quantity of food intake during TRE remained consistent with participants’ habitual diets. No adverse or unintended effects occurred in either group.

### 3.3. Menopausal Symptoms and Quality of Life

At baseline, the total scores in the Menopause Rating Scale (MRS) and all subdomain scores were similar between the exercise group and the combination group. After 12 weeks, the exercise group showed no significant changes in MRS outcomes (Table 1). The combination group exhibited significant decreases in the total MRS scores (moderate effect size), as well as in the psychological (moderate effect size) and somatic (moderate effect size) subdomains (Table 1). There was no significant change in the MRS urogenital score for the combination group after the intervention (Table 1).

To test the research question, regression models were estimated, with the dependent variables defined as the differences between baseline and final values of questionnaire assessment outcome measures. Undergoing TRE among the women studied showed statistically significant effect on reducing the total MRS score (*p* = 0.008). When combined with exercise, TRE led to a decrease in the mean MRS score by 4.92 units over the observation period, compared to the exercise group. The change in LMR also demonstrated significant effect (at 0.03 level) on the change in total MRS: an increase of one unit in LMR was associated with a 3.19-unit increase in the mean total MRS score. Other explanatory variables—including age, BMI, and the interaction between TRE and change in LMR—were not statistically significant. This indicates that the observed effects of TRE and LMR change on total MRS were independent of these factors. TRE combined with exercise also had a significant effect on two MRS subdomains: psychological (*p* = 0.009) and somatic (*p* = 0.007). It reduces the mean scores in these domains by 2.69 and 1.56 points, respectively. No significant effect of TRE was observed in the urogenital subdomain score. In the subdomain-specific models, none of the control variables reached statistical significance, except for change in LMR in the psychological subdomain model (*p* = 0.012).

The values for the MENQOL vasomotor, psychosocial, and physical domains were similar between the exercise group and the combination group at baseline. The average score of the sexual domain in MENQOL was significantly lower in the combination group compared to the exercise group at baseline (*p* = 0.013). After 12 weeks, there were no significant changes in any MENQOL domains for the exercise group (Table 1). In the combination group, significant decreases were observed in the MENQOL psychosocial (small effect size) and physical (moderate effect size) domains, with no changes in the vasomotor and sexual domains (Table 1). Regression models show that the effects of TRE on the MENQOL dependent variable varied across subdomains. Statistically significant effects were observed for the psychosocial subdomain (*p* = 0.002) and physical subdomain (*p* = 0.013), while no significant effects were found for the vasomotor and sexual subdomains. All estimated coefficients were positive. Considering those statistically significant only, one could claim that TRE combined with exercise led to a greater decrease in mean MENQOL scores by 11.85 (psychosocial subdomain) and by 15.85 (physical subdomain) than exercise only. As with most of the models reported here, other regressors were not statistically significant, with one exception. In the physical subdomain model the interaction between change in LMR an TRE was significant at the 0.01 level. This suggests a different effect of LMR change between the combination group and the exercise group: the impact of LMR change on the physical subdomain was 21.25 units lower in the TRE group compared to the exercise group.

In regressions based on individual cross-sectional data-as in this study—R-squared values rarely exceed 0.5. This pattern holds true here as well. The highest R-squared values, exceeding 0.2, were found for the psychological MRS and the psychosocial and physical subdomains of MENQOL. There is no absolute lower threshold for R-squared that renders a model invalid, as long as the estimate itself is statistically significant. This criterion, however, was not met in regressions for the somatic and urogenital MRS subdomains, as well as for the vasomotor and sexual MENQOL subdomains. That said, a non-significant R-squared does not necessarily imply that the effects of individual variables are also non-significant. To assess the size of such effects, partial correlation coefficients can be used. These measure the relative increase in the sum of squared residuals when a specific variable (in this case, TRE) is removed from the model. The greater the reduction in the proportion of explained variance, the larger the effect of the removed variable. Squared partial correlation.

Coefficients for all variables are presented in Table 2. These can be interpreted as the squared linear correlation between TRE and the dependent variable, controlling for all other variables in the model.

### 3.4. Life Satisfaction, General Health, Fatigue, Sleep Quality

At baseline, there was no significant difference between the exercise and combination groups in life satisfaction (Satisfaction with Life Scale, SWLS), general health (General Health Questionnaire, GHQ-12), fatigue (Fatigue Assessment Scale, FAS: total score, mental and physical subdomains), or sleep quality (Single-Item Sleep Quality Scale, SQS). After 12 weeks, no significant changes were observed in life satisfaction, general health, mental or physical fatigue, or sleep quality in the exercise group, although there was a significant decrease in the total fatigue (small effect size) (Table 1). In the combination group, there was a significant increase in life satisfaction (small effect size) and significant decreases in general health (moderate effect size) and mental fatigue (moderate effect size) after 12 weeks (Table 1). No significant changes were observed in total fatigue, mental fatigue, or sleep quality in the combination group after the intervention (Table 1). Regression models show that significant effects of TRE were observed for life satisfaction and general health (both *p* < 0.01), as well as for mental fatigue (*p* = 0.02) (Table 2). The effect on life satisfaction was negative, indicating that TRE increased the mean SWLS score by 3.6 points (Table 2). In contrast, the effects on general health and mental fatigue were positive, suggesting that TRE reduced the mean GHQ-12 score by 6.02 points and the mental FAS score by 2.41 points (Table 2). No statistically significant effects of TRE were found for total FAS score, physical fatigue, or sleep quality (Table 2). Squared partial correlation coefficients for all variables are presented in Table 2.

## 4. Discussion

This study is, to our knowledge, the first to explore the joint effects of intermittent fasting and exercise on menopausal symptoms and quality of life in women during menopause. The aim of the study was to determine whether implementing TRE alongside exercise, compared with exercise alone, would produce additional benefits for this population. The authors hypothesized that combination of TRE and exercise would yield greater improvements in both menopausal symptoms and quality of life, and the results of the study confirmed this hypothesis. The main finding was that TRE combined with exercise decreased the severity of menopausal symptoms and improved quality of life more compared to exercise alone.

Duijts et al. [44] examined the impact of a 12-week home-based physical exercise program (2.5–3 h per week) on menopausal symptoms such as hot flashes and night sweats in breast cancer patients experiencing treatment-induced menopause, using the Hot Flush Rating Scale. No significant changes were found in this study. Similarly, there was no significant reduction in the vasomotor subscale of the MENQOL questionnaire in this study. Costa et al. [45] evaluated the effects of 10 weeks of isoflavone supplementation combined with aerobic and resistance training (45 min, three times a week) on menopausal symptoms in postmenopausal women, using the Menopause Rating Scale. This study reported significant decreases in total MRS scores and all subscales (somatic, urogenital, psychological) in the group that received exercise training and placebo. In contrast, the current study found no significant changes in any MRS subscale in the exercise group. However, the women in this study were younger and included those who were perimenopausal and menopausal, not just postmenopausal. Their baseline MRS scores were lower, which may explain why significant symptom reduction was not observed, as the initial symptom levels were not high. Additionally, no placebo was administered to the exercise group, which might explain the lack of additional effects.

Reed et al. [46] investigated the impact of individualized facility-based aerobic exercise training performed three times per week on the menopausal quality of life, as measured by the MENQOL questionnaire, in perimenopausal and postmenopausal women. While there were no significant improvements in the MENQOL total score or in the vasomotor, psychosocial, and sexual domains, exercise significantly benefited the MENQOL physical domain score. In this study, although the exercise group did not show significant improvements in MENQOL scores, the combination group exhibited significantly greater improvement in MENQOL psychosocial and physical subdomains, indicating that combining TRE with exercise results in superior improvements in menopausal quality of life compared to exercise alone.

The exploratory analysis by Kahleova et al. [47] indicated that a 12-week specific diet (a low-fat, vegan diet including 86 g of soybeans per day) may reduce the occurrence of hot flashes, with changes in the abundance of certain gut microbiota species associated with this reduction. Furthermore, it is known that IF influences gut microbiota [48,49]. It has been proven that IF alters the gut microbiome, increasing bacterial richness in animal models [50]. Additionally, an 8-week ‘2 day’ modified IF regimen in patients with metabolic syndrome led to significant positive changes in gut microbiota communities [51]. In the study conducted by Czerwińska-Ledwig et al. [52], patients with multiple myeloma underwent a 6-week Nordic Walking program—performed three times per week at moderate intensity—combined with a 10/14 time-restricted eating regimen, which resulted in increased biodiversity and a taxonomic rearrangement of gut microbiota species. Given that IF and IF combined with exercise positively affect gut microbiota and improvements in gut microbiota are linked to reduced severity of hot flashes, the observed improvements in menopausal symptoms using MRS in the combination group of this study might be explained by potential alterations in gut microbiota. Nonetheless, future studies should investigate combined TRE and exercise interventions while controlling for gut microbiota alterations to better understand the mechanisms underlying the reduction of menopausal symptoms.

The study demonstrated that participation in the combination group—engaging in both exercise and time-restricted eating—was associated with a significantly greater improvement in the physical domain of menopause-related quality of life (MENQOL—physical domain) compared to the exercise group. Importantly, the interaction between the change in the lymphocyte-to-monocyte ratio (LMR) and assignment to the TRE group was statistically significant. This finding suggests that the association between LMR change and improvement in physical menopausal quality of life differed between the two groups: the impact of LMR change on outcomes was significantly weaker in the TRE group than in the exercise group. In other words, while an increase in LMR tended to correlate with worsening of physical MENQOL scores in the exercise group, this relationship was attenuated in participants undergoing exercise and TRE. Moreover, the main effect of LMR change was also evident in relation to menopausal symptoms assessed by the MRS. A statistically significant positive association was found between an increase in LMR and an increase in the total MRS scores and psychological MRS subdomain scores. Simultaneously, TRE combined with exercise led to a statistically significant decrease in total MRS, as well as in its psychological and somatic subdomains. Notably, other explanatory variables such as age, BMI, and the interaction between TRE and LMR were not significant in the MRS model, indicating that the observed effects of TRE and LMR change on total MRS were independent of these factors. While LMR has been recognized as a biomarker of systemic inflammation—known to be elevated, for example, in rheumatoid arthritis and correlated with classic inflammatory markers such as erythrocyte sedimentation rate (ESR) and rheumatoid factors (RF) [53]—its role in the current study appears more nuanced. In a study by Faris et al. [54], intermittent fasting (Ramadan) reduced pro-inflammatory cytokines and altered lymphocyte and monocyte counts. However, in the present study, the magnitude of the LMR effect was diminished in the TRE group, suggesting that the benefits of TRE may not be mediated solely through inflammatory reduction. These findings should be interpreted in the broader context of evidence suggesting that menopausal symptoms are linked to low-grade systemic inflammation [55,56]. Taken together, the results indicate that higher post-intervention systemic inflammation—as reflected by increased LMR—was associated with worse menopausal symptoms, particularly in the absence of TRE. However, TRE appeared to buffer this association in the context of menopause-related physical quality of life and exerted a direct, beneficial effect on both MRS and MENQOL outcomes. This observation may imply that the beneficial effects of TRE on menopausal symptoms are not solely driven by reductions in systemic inflammation as measured by LMR. Rather, TRE may act through additional or alternative pathways, potentially independent of changes in immune-inflammatory status.

Reduction in the severity of menopausal symptoms may occur in response to changes in sex hormone levels [57]. Three studies have examined the effects of fasting strategies on sex steroid levels in women. Harvie et al. [58] found no changes in the levels of prolactin, testosterone, and androstenedione after 24 weeks of the 5:2 diet (2 days of 500 kcal intake and 5 days of ad libitum calorie intake) in premenopausal women. Similarly, Li et al. [59] reported no changes in follicle-stimulating hormone (FSH), luteinizing hormone (LH), or testosterone after 5 weeks of 8-h TRE in premenopausal women with polycystic ovary syndrome (PCOS). Kalam et al. [60] also observed no changes in sex hormone-binding globulin (SHGB), testosterone, androstenedione in both premenopausal and postmenopausal women, and no changes in estrogen levels in postmenopausal women. Since these studies found no change in sex hormones following fasting strategies, the positive effect of combining TRE and exercise on the severity of menopausal symptoms measured with MRS in this study is likely not due to changes in sex hormone levels among participants. Nonetheless, future studies should investigate combined TRE and exercise interventions while controlling for sex hormone levels to better understand the mechanisms underlying the reduction of menopausal symptoms.

In the present study, no significant effects were observed on the urogenital (MRS) or sexual (MENQOL) subdomains in either group, likely because the interventions did not include targeted pelvic floor muscle exercises. Previous research suggests that such exercises are necessary to improve urogenital and sexual function in postmenopausal women [61,62,63]. Another possible explanation is the relatively short intervention period (12 weeks), which may have been insufficient to induce measurable changes in these outcomes. These findings underline the need for longer or more tailored interventions—such as combining TRE and strength and endurance training programs with specific pelvic floor muscles exercises or hormonal support programs—to effectively address urogenital and sexual domains of menopausal symptoms and quality of life.

Life satisfaction improved only in the combination group, highlighting the potential importance of combining exercise with dietary interventions such as TRE. While exercise alone has limited effects on life satisfaction [64], combining exercise with dietary therapy appears to enhance well-being [65]. Consistently, mental health (GHQ-12) improved more in the combination group than in the exercise group, supporting previous research suggesting that dietary interventions alongside exercise can enhance psychological well-being [11].

Mental fatigue decreased significantly only in the combination group, suggesting that TRE with exercise specifically targets mental aspects of fatigue. No effect was observed on physical fatigue in either group. Notably, previous research indicates that an early TRE (eTRE) regimen with a fixed eating window may yield more pronounced benefits on fatigue reduction than self-selected TRE schedules but may also negatively affect sleep by reducing duration and efficiency [9]. In contrast, the present study found no effects on sleep, suggesting that combining TRE with exercise or using non-early eating windows may mitigate such sleep disturbances but may not be sufficient to produce measurable improvements in sleep quality. Together, these results suggest that future interventions should consider extending the duration or incorporating complementary behavioral components, such as sleep hygiene education, to achieve broader benefits. Additionally, using multi-component questionnaires rather than single-item tools may provide a more comprehensive assessment of sleep quality.

The findings of this study may be applicable to diverse populations and cultural backgrounds in terms of general well-being. Importantly, the results remained significant despite relatively low adherence to the diet protocol. Fasting is part of many religious and ethnic practices, yet benefits were evident even among participants whose usual cultural habits did not include fasting. This suggests that fasting interventions may confer health benefits across different cultural and dietary contexts, although adherence and outcomes may vary depending on individual and sociocultural factors.

The study has several limitations. Sex hormone levels were not measured, and the phase of menopause was self-reported. Measuring these hormone levels could have clarified the relationship between exercise and diet and the alleviation of menopausal symptoms, while also providing an objective measure of the menopausal phase. Although a food frequency questionnaire was used and meal timing recorded, detailed data on energy intake and nutrient composition were unavailable, limiting the assessment of how energy restriction may have influenced the study outcomes. Future studies should include dietary tracking or objective measures to more accurately determine the effects of the fasting intervention. The sample size was also quite limited, and there was notably higher loss-to-follow-up in the combination group than in the exercise group. The trial was conducted in different seasons, which may have affected habitual physical activity and represents an additional limitation. Moreover, the study used a quasi-randomized design, with group allocation partly determined by participant preference. This approach may have introduced selection bias. Future research with larger samples and more diverse study designs—such as including a fasting-only group and a control group—is needed to identify the optimal durations of fasting and exercise interventions for enhancing quality of life and alleviating menopausal symptoms.

## 5. Conclusions

We demonstrated that combining time-restricted eating with exercise provides greater relief from menopausal symptoms and mental fatigue, and improves quality of life, psychological well-being, and life satisfaction compared to exercise alone. TRE did not further enhance urogenital menopausal symptoms and sexual quality of life. This combined approach is a cost-effective, accessible strategy with minimal contraindications or adverse effects, offering a promising way to prevent menopausal symptoms and maintain overall well-being. These findings provide valuable insight for developing lifestyle modification guidelines tailored to menopausal women.

## Figures and Tables

**Table 1:** The effect of diet and/or exercise on outcome measures.

	Exercise Group (*n* = 30)		*p,* r	Combination Group (*n* = 24)		*p,* r
	Baseline	12 Weeks		Baseline	12 Weeks	
MRS total	14.20 ± 5.83, 13.50 (10.00, 18.00)	13.60 ± 6.05, 12.00 (10.00, 19.00)	0.665, 0.079	15.00 ± 6.09, 14.00 (10.50, 20.50)	10.83 ± 5.64, 10.50 (7.00, 14.50)	0.003 *, 0.598
MRS psychological	5.60 ± 3.37, 5.00 (3.00, 8.00)	5.00 ± 3.29, 4.00 (2.00, 8.00)	0.434, 0.143	6.13 ± 2.97, 6.50 (4.00, 8.00)	3.75 ± 2.45, 4.00 (2.50, 5.00)	0.004 *, 0.596
MRS somatic	4.67 ± 2.20, 4.50 (3.00, 6.00)	4.90 ± 2.07, 5.00 (4.00, 6.00)	0.495, 0.124	5.17 ± 2.14, 5.00 (4.00, 6.50)	4.17 ± 2.16, 4.00 (3.00, 5.50)	0.009 *, 0.534
MRS urogenital	3.93 ± 1.93, 4.00 (3.00, 6.00)	3.70 ± 2.22, 4.00 (2.00, 5.00)	0.641, 0.085	3.71 ± 2.66, 3.50 (2.00, 6.00)	2.92 ± 2.36, 2.50 (1.50, 3.00)	0.130, 0.309
MENQOL vasomotor	9.60 ± 5.73, 8.00 (5.00, 13.00)	9.03 ± 6.05, 7.00 (4.00, 12.00)	0.187, 0.241	7.79 ± 5.10, 6.50 (3.00, 9.50)	5.92 ± 3.31, 5.00 (3.00, 7.00)	0.063, 0.380
MENQOL psychosocial	27.33 ± 11.57, 24.50 (17.00, 39.00)	26.10 ± 11.69, 23.50 (15.00, 37.00)	0.585, 0.100	28.17 ± 9.74, 25.50 (21.50, 36.00)	20.63 ± 10.27, 18.00 (14.50, 25.00)	0.021 *, 0.472
MENQOL physical	64.03 ± 20.94, 63.00 (47.00, 80.00)	57.27 ± 25.68, 53.00 (33.00, 78.00)	0.066, 0.336	59.38 ± 16.79, 61.50 (44.00, 71.50)	46.25 ± 19.14, 39.00 (30.00, 61.00)	0.010 *, 0.525
MENQOL sexual	11.70 ± 6.27, 10.00 (7.00, 17.00)	10.47 ± 5.81, 10.00 (6.00, 12.00)	0.219, 0.225	7.63 ± 4.73, 6.50 (3.00, 10.50)	7.29 ± 5.67, 5.00 (3.00, 9.50)	0.660, 0.090
SWLS	23.73 ± 4.62, 23.00 (20.00, 27.00)	23.47 ± 5.15, 24.00 (19.00, 28.00)	0.517, 0.118	23.04 ± 4.66, 24.00 (20.50, 26.50)	25.04 ± 4.59, 26.50 (23.50, 28.00)	0.014 *, 0.499
FAS total	25.77 ± 6.94, 23.50 (20.00, 32.00)	23.40 ± 6.30, 21.50 (19.00, 29.00)	0.038 *, 0.378	25.63 ± 6.19, 24.50 (22.00, 30.00)	23.04 ± 3.93, 23.50 (21.00, 25.00)	0.117, 0.320
FAS mental	12.53 ± 3.70, 11.50 (10.00, 16.00)	11.60 ± 3.38, 11.00 (9.00, 14.00)	0.155, 0.260	12.21 ± 3.34, 12.00 (10.00, 14.00)	10.00 ± 2.62, 9.50 (8.00, 12.50)	0.002 *, 0.620
FAS physical	13.23 ± 3.79, 13.00 (10.00, 16.00)	11.80 ± 3.58, 11.00 (9.00, 15.00)	0.050, 0.357	13.42 ± 3.49, 14.00 (10.50, 16.00)	13.04 ± 2.91, 14.00 (11.50, 14.50)	0.715, 0.074
GHQ-12	25.87 ± 4.13, 25.00 (23.00, 29.00)	25.07 ± 5.87, 24.50 (22.00, 29.00)	0.353, 0.169	27.25 ± 5.74, 26.50 (23.00, 31.00)	22.67 ± 4.34, 22.50 (20.50, 24.50)	0.002 *, 0.636
SQS	5.70 ± 1.99, 6.00 (4.00, 7.00)	5.93 ± 5.15, 6.00 (19.00, 28.00)	0.484, 0.128	5.00 ± 1.67, 5.50 (4.00, 6.00)	5.81 ± 2.07, 6.00 (4.50, 7.00)	0.177, 0.337
LMR	4.25 ± 1.13, 4.09 (3.45, 5.02)	4.10 ± 0.97, 4.14 (3.63, 4.78)	0.206, 0.258	4.00 ± 1.37 3.92 (3.8, 4.73)	4.44 ± 1.39 4.44 (3.9, 5.01)	0.006 *, 0.560
BMI	26.17 ± 5.43, 25.40 (21.40, 29.00)	25.93 ± 5.38, 25.15 (21.20, 29.10)	0.294, 0.191	27.85 ± 5.46 26.75 (23.25, 32.60)	27.18 ± 5.22 26.10 (22.50, 31.40)	0.001 *, 0.652

Note: Values are means ± SD and medians (quartiles); *—*p* < 0.05; r 0.50–0.80 indicating moderate effect or r > 0.80 indicating large effect. Abbreviations: MRS—Menopause Rating Scale, MENQOL—Menopause-Specific Quality of Life Questionnaire, SWLS—Satisfaction with Life Scale, FAS—Fatigue Assessment Scale, GHQ-12—General Health Questionnaire, SQS—Single-Item Sleep Quality Scale, LMR—lymphocyte-to-monocyte ratio, BMI—body mass index.

**Table 2:** The effect of TRE on outcome measures: estimates of regression models.

Dependent Variables	Independent Variables					R-Squared	Partial Correlation
	TRE	Age	Initial BMI	Change in LMR	LMR × TRE (Interaction)		
MRS total	4.922	−0.036	−0.057	−3.192	1.615	0.194	0.136
	0.008 *	0.838	0.703	0.031 *	0.487	0.057	0.008 *
MRS psychological	2.689	0.06	−0.037	−2.081	0.45	0.236	0.134
	0.009 *	0.545	0.659	0.012 *	0.725	0.021 *	0.009 *
MRS somatic	1.564	−0.032	−0.007	−0.285	−0.139	0.150	0.144
	0.007 *	0.556	0.883	0.523	0.846	0.156	0.007 *
MRS urogenital	0.669	−0.063	−0.014	−0.825	1.304	0.080	0.017
	0.366	0.386	0.823	0.168	0.174	0.529	0.366
MENQOL vasomotor	2.608	−0.224	0.074	−0.319	−1.375	0.079	0.052
	0.111	0.166	0.586	0.806	0.510	0.539	0.111
MENQOL psychosocial ^1^	11.853	−0.533	0.219	−2.221	−6.767	0.273	0.186
	0.002 *	0.144	0.472	0.507	0.221	0.016 *	0.002 *
MENQOL physical	15.846	−1.077	−0.113	5.298	−21.248	0.216	0.122
	0.013 *	0.082	0.827	0.287	0.010 *	0.034 *	0.013 *
MENQOL sexual ^2^	0.544	3.223	−0.019	−1.158	−0.859	0.118	0.003
	0.711	0.274	0.878	0.332	0.651	0.407	0.711
SWLS	−3.597	0.208	−0.011	1.107	0.262	0.211	0.181
	0.002 *	0.063	0.904	0.219	0.855	0.038	0.002 *
FAS total	2.931	−0.197	−0.136	−1.737	−2.072	0.140	0.048
	0.125	0.293	0.392	0.256	0.397	0.189	0.125
FAS mental	2.406	−0.005	−0.021	−0.798	−1.390	0.144	0.104
	0.022 *	0.957	0.806	0.335	0.295	0.174	0.022 *
FAS physical	0.525	−0.192	−0.115	−0.939	−0.681	0.127	0.003
	0.694	0.150	0.306	0.383	0.692	0.243	0.694
GHQ-12	6.022	−0.298	−0.005	−1.887	−0.651	0.177	0.156
	0.004 *	0.142	0.978	0.251	0.804	0.087	0.005 *
SQS	−0.553	−0.062	0.053	0.082	0.016	0.038	0.011
	0.508	0.457	0.481	0.895	0.988	0.902	0.508

Note: Coefficient estimates in the first row, *p*-values in the second row; *—*p* < 0.05; ^1^ diLMR squared added, ^2^ age squared added (estimates not reported). Abbreviations: MRS—Menopause Rating Scale, MENQOL—Menopause-Specific Quality of Life Questionnaire, SWLS—Satisfaction with Life Scale, FAS—Fatigue Assessment Scale, GHQ-12—General Health Questionnaire, SQS—Single-Item Sleep Quality Scale, TRE—time-restricted eating, BMI—body mass index, LMR—lymphocyte-to-monocyte ratio.

## Data Availability

The raw data supporting the conclusions of this article will be made available by the authors on request.

## Abbreviations

The following abbreviations are used in this manuscript:

1RM	One-repetition maximum
AMPK	Adenosine monophosphate-activated protein kinase
BMI	Body mass index
ES	Effect size
eTRE	Early time-restricted eating
FAS	Fatigue Assessment Scale
GHQ-12	General Health Questionnaire
IF	Intermittent fasting
KomPAN	Questionnaire for Dietary Habits, Lifestyle, and Nutrition Knowledge Assessment
LMR	Lymphocyte-to-monocyte ratio
MENQOL	Menopause-Specific Quality of Life Questionnaire
MRS	Menopause Rating Scale
mTOR	Mechanistic target of rapamycin
SQS	Single-Item Sleep Quality Scale
SWLS	Satisfaction with Life Scale
TRE	Time-restricted eating
ULK1	Unc-51-like kinase 1

## References

[1] Hybholt M. (2022). Psychological and Social Health Outcomes of Physical Activity around Menopause: A Scoping Review of Research. Maturitas.

[2] Lambrinoudaki I., Armeni E., Goulis D., Bretz S., Ceausu I., Durmusoglu F., Erkkola R., Fistonic I., Gambacciani M., Geukes M. (2022). Menopause, Wellbeing and Health: A Care Pathway from the European Menopause and Andropause Society. Maturitas.

[3] Duralde E.R., Sobel T.H., Manson J.E. (2023). Management of Perimenopausal and Menopausal Symptoms. BMJ.

[4] Madsen T.E., Sobel T., Negash S., Shrout Allen T., Stefanick M.L., Manson J.E., Allison M. (2023). A Review of Hormone and Non-Hormonal Therapy Options for the Treatment of Menopause. Int. J. Womens Health.

[5] Kulak A., Toros T., Ogras E.B., Etiler I.E., Bagci E., Gokyurek B., Bilgin U. (2023). The Impact of Sustainable Exercise on Self-Efficacy and Life Satisfaction in Women before and after Menopause. Behav. Sci..

[6] Nguyen T.M., Do T.T.T., Tran T.N., Kim J.H. (2020). Exercise and Quality of Life in Women with Menopausal Symptoms: A Systematic Review and Meta-Analysis of Randomized Controlled Trials. Int. J. Environ. Res. Public Health.

[7] Tandon V.R., Sharma S., Mahajan A., Mahajan A., Tandon A. (2022). Menopause and Sleep Disorders. J. Midlife Health.

[8] El Hajj A., Wardy N., Haidar S., Bourgi D., Haddad M.E., Chammas D.E., El Osta N., Rabbaa Khabbaz L., Papazian T. (2020). Menopausal Symptoms, Physical Activity Level and Quality of Life of Women Living in the Mediterranean Region. PLoS ONE.

[9] Steger F.L., Jamshed H., Bryan D.R., Richman J.S., Warriner A.H., Hanick C.J., Martin C.K., Salvy S.-J., Peterson C.M. (2023). Early Time-Restricted Eating Affects Weight, Metabolic Health, Mood, and Sleep in Adherent Completers: A Secondary Analysis. Obesity.

[10] Grigolon R.B., Ceolin G., Deng Y., Bambokian A., Koning E., Fabe J., Lima M., Gerchman F., Soares C.N., Brietzke E. (2023). Effects of Nutritional Interventions on the Severity of Depressive and Anxiety Symptoms of Women in the Menopausal Transition and Menopause: A Systematic Review, Meta-Analysis, and Meta-Regression. Menopause.

[11] Choda N., Wakai K., Naito M., Imaeda N., Goto C., Maruyama K., Kadomatsu Y., Tsukamoto M., Sasakabe T., Kubo Y. (2020). Associations between Diet and Mental Health Using the 12-Item General Health Questionnaire: Cross-Sectional and Prospective Analyses from the Japan Multi-Institutional Collaborative Cohort Study. Nutr. J..

[12] Hao S., Tan S., Li J., Li W., Li J., Cai X., Hong Z. (2021). Dietary and Exercise Interventions for Perimenopausal Women: A Health Status Impact Study. Front. Nutr..

[13] Chaix A., Rynders C.A. (2022). Time Restricted Feeding plus Exercise: Could Two Be Better than One for Metabolic Health?. J. Physiol..

[14] Vasim I., Majeed C.N., DeBoer M.D. (2022). Intermittent Fasting and Metabolic Health. Nutrients.

[15] Gudden J., Arias Vasquez A., Bloemendaal M. (2021). The Effects of Intermittent Fasting on Brain and Cognitive Function. Nutrients.

[16] Jamshed H., Steger F.L., Bryan D.R., Richman J.S., Warriner A.H., Hanick C.J., Martin C.K., Salvy S.-J., Peterson C.M. (2022). Effectiveness of Early Time-Restricted Eating for Weight Loss, Fat Loss, and Cardiometabolic Health in Adults with Obesity: A Randomized Clinical Trial. JAMA Intern. Med..

[17] Longo V.D., Mattson M.P. (2014). Fasting: Molecular Mechanisms and Clinical Applications. Cell Metab..

[18] Germic N., Frangez Z., Yousefi S., Simon H.-U. (2019). Regulation of the Innate Immune System by Autophagy: Monocytes, Macrophages, Dendritic Cells and Antigen Presentation. Cell Death Differ..

[19] Bhattacharya A., Eissa N.T. (2015). Autophagy as a Stress Response Pathway in the Immune System. Int. Rev. Immunol..

[20] Khater S.I., Shalabi M., Alammash B.B., Alrais A.I., Al-ahmadi D., Alqahtani L.S., Khamis T., Abdelaziz S., Aldawy K. (2023). Autophagy Characteristics of Phytoestrogens in Management and Prevention of Diseases: A Narrative Review of in-Vivo and in-Vitro Studies. J. Adv. Vet. Anim. Res..

[21] van Niekerk G., Hattingh S.M., Engelbrecht A.-M. (2016). Enhanced Therapeutic Efficacy in Cancer Patients by Short-Term Fasting: The Autophagy Connection. Front. Oncol..

[22] Hofer S.J., Carmona-Gutierrez D., Mueller M.I., Madeo F. (2022). The Ups and Downs of Caloric Restriction and Fasting: From Molecular Effects to Clinical Application. EMBO Mol. Med..

[23] Hannan M.A., Rahman M.A., Rahman M.S., Sohag A.A.M., Dash R., Hossain K.S., Farjana M., Uddin M.J. (2020). Intermittent Fasting, a Possible Priming Tool for Host Defense against SARS-CoV-2 Infection: Crosstalk among Calorie Restriction, Autophagy and Immune Response. Immunol. Lett..

[24] Bagherniya M., Butler A.E., Barreto G.E., Sahebkar A. (2018). The Effect of Fasting or Calorie Restriction on Autophagy Induction: A Review of the Literature. Ageing Res. Rev..

[25] Dengjel J., Schoor O., Fischer R., Reich M., Kraus M., Müller M., Kreymborg K., Altenberend F., Brandenburg J., Kalbacher H. (2005). Autophagy Promotes MHC Class II Presentation of Peptides from Intracellular Source Proteins. Proc. Natl. Acad. Sci. USA.

[26] Jordan S., Tung N., Casanova-Acebes M., Chang C., Cantoni C., Zhang D., Wirtz T.H., Naik S., Rose S.A., Brocker C.N. (2019). Dietary Intake Regulates the Circulating Inflammatory Monocyte Pool. Cell.

[27] Congdon E.E. (2018). Sex Differences in Autophagy Contribute to Female Vulnerability in Alzheimer’s Disease. Front. Neurosci..

[28] Anton S.D., Moehl K., Donahoo W.T., Marosi K., Lee S.A., Mainous A.G., Leeuwenburgh C., Mattson M.P. (2018). Flipping the Metabolic Switch: Understanding and Applying the Health Benefits of Fasting. Obesity.

[29] Hao S., Tan S., Li J., Li W., Li J., Liu Y., Hong Z. (2022). The Effect of Diet and Exercise on Climacteric Symptomatology. Asia Pac. J. Clin. Nutr..

[30] Ashtary-Larky D., Bagheri R., Tinsley G.M., Asbaghi O., Paoli A., Moro T. (2021). Effects of Intermittent Fasting Combined with Resistance Training on Body Composition: A Systematic Review and Meta-Analysis. Physiol. Behav..

[31] Keenan S., Cooke M.B., Belski R. (2020). The Effects of Intermittent Fasting Combined with Resistance Training on Lean Body Mass: A Systematic Review of Human Studies. Nutrients.

[32] Reddy B.L., Reddy V.S., Saier M.H. (2024). Health Benefits of Intermittent Fasting. Microb. Physiol..

[33] Armstrong T., Bull F. (2006). Development of the World Health Organization Global Physical Activity Questionnaire (GPAQ). J. Public Health.

[34] Sawicka-Gutaj N., Gruszczyński D., Guzik P., Mostowska A., Walkowiak J. (2022). Publication Ethics of Human Studies in the Light of the Declaration of Helsinki—A Mini-Review. J. Med. Sci..

[35] Heinemann L.A.J., Potthoff P., Schneider H.P.G. (2003). International Versions of the Menopause Rating Scale (MRS). Health Qual. Life Outcomes.

[36] Hilditch J.R., Lewis J., Peter A., van Maris B., Ross A., Franssen E., Guyatt G.H., Norton P.G., Dunn E. (1996). A Menopause-Specific Quality of Life Questionnaire: Development and Psychometric Properties. Maturitas.

[37] Diener E., Emmons R.A., Larsen R.J., Griffin S. (1985). The Satisfaction With Life Scale. J. Pers. Assess..

[38] De Vries J., Michielsen H., Van Heck G.L. (2003). Assessment of Fatigue among Working People: A Comparison of Six Questionnaires. Occup. Environ. Med..

[39] Goldberg D.P., Gater R., Sartorius N., Ustun T.B., Piccinelli M., Gureje O., Rutter C. (1997). The Validity of Two Versions of the GHQ in the WHO Study of Mental Illness in General Health Care. Psychol. Med..

[40] Snyder E., Cai B., DeMuro C., Morrison M.F., Ball W. (2018). A New Single-Item Sleep Quality Scale: Results of Psychometric Evaluation in Patients With Chronic Primary Insomnia and Depression. J. Clin. Sleep Med..

[41] Kowalkowska J., Wadolowska L., Czarnocinska J., Czlapka-Matyasik M., Galinski G., Jezewska-Zychowicz M., Bronkowska M., Dlugosz A., Loboda D., Wyka J. (2018). Reproducibility of a Questionnaire for Dietary Habits, Lifestyle and Nutrition Knowledge Assessment (KomPAN) in Polish Adolescents and Adults. Nutrients.

[42] Rosenthal R. (1994). Parametric Measures of Effect Size. The Handbook of Research Synthesis.

[43] Lakens D. (2013). Calculating and Reporting Effect Sizes to Facilitate Cumulative Science: A Practical Primer for t-Tests and ANOVAs. Front. Psychol..

[44] Duijts S.F.A., van Beurden M., Oldenburg H.S.A., Hunter M.S., Kieffer J.M., Stuiver M.M., Gerritsma M.A., Menke-Pluymers M.B.E., Plaisier P.W., Rijna H. (2012). Efficacy of Cognitive Behavioral Therapy and Physical Exercise in Alleviating Treatment-Induced Menopausal Symptoms in Patients With Breast Cancer: Results of a Randomized, Controlled, Multicenter Trial. J. Clin. Oncol..

[45] Costa J.G., Giolo J.G., Mariano I.M., Batista J.P., Ribeiro A.L.A., Souza T.C.F., de Oliveira E.P., Resende A.P.M., Puga G.M. (2017). Combined exercise training reduces climacteric symptoms without the additive effects of isoflavone supplementation: A clinical, controlled, randomised, double-blind study. Nutr. Health.

[46] Reed S.D., Guthrie K.A., Newton K.M., Anderson G.L., Booth-Laforce C., Caan B., Carpenter J.S., Cohen L.S., Dunn A.L., Ensrud K.E. (2014). Menopausal Quality of Life: A RCT of Yoga, Exercise and Omega-3 Supplements. Am. J. Obstet. Gynecol..

[47] Kahleova H., Holtz D.N., Strom N., La Reau A., Kolipaka S., Schmidt N., Hata E., Znayenko-Miller T., Holubkov R., Barnard N.D. (2023). A Dietary Intervention for Postmenopausal Hot Flashes: A Potential Role of Gut Microbiome. An Exploratory Analysis. Complement. Ther. Med..

[48] Paukkonen I., Törrönen E.-N., Lok J., Schwab U., El-Nezami H. (2024). The Impact of Intermittent Fasting on Gut Microbiota: A Systematic Review of Human Studies. Front. Nutr..

[49] Cadena-Ullauri S., Guevara-Ramírez P., Ruiz-Pozo V.A., Tamayo-Trujillo R., Paz-Cruz E., Zambrano-Villacres R., Simancas-Racines D., Zambrano A.K. (2024). The Effect of Intermittent Fasting on Microbiota as a Therapeutic Approach in Obesity. Front. Nutr..

[50] Cignarella F., Cantoni C., Ghezzi L., Salter A., Dorsett Y., Chen L., Phillips D., Weinstock G.M., Fontana L., Cross A.H. (2018). Intermittent Fasting Confers Protection in CNS Autoimmunity by Altering the Gut Microbiota. Cell Metab..

[51] Guo Y., Luo S., Ye Y., Yin S., Fan J., Xia M. (2021). Intermittent Fasting Improves Cardiometabolic Risk Factors and Alters Gut Microbiota in Metabolic Syndrome Patients. J. Clin. Endocrinol. Metab..

[52] Czerwińska-Ledwig O., Nowak-Zaleska A., Żychowska M., Meyza K., Pałka T., Dzidek A., Szlachetka A., Jurczyszyn A., Piotrowska A. (2025). The Positive Effects of Training and Time-Restricted Eating in Gut Microbiota Biodiversity in Patients with Multiple Myeloma. Nutrients.

[53] Obaid J.M.A.S., Almjydy M.M.A., Garban M.A.Q., Al-hebari F.S.Q., Al-washah N.A.H. (2023). Neutrophil-to-monocyte Ratio Is the Better New Inflammatory Marker Associated with Rheumatoid Arthritis Activity. Health Sci. Rep..

[54] Faris M.A.-I.E., Kacimi S., Al-Kurd R.A., Fararjeh M.A., Bustanji Y.K., Mohammad M.K., Salem M.L. (2012). Intermittent Fasting during Ramadan Attenuates Proinflammatory Cytokines and Immune Cells in Healthy Subjects. Nutr. Res..

[55] Korpe B., Kose C., Keskin H.L. (2024). Systemic Inflammation and Menopausal Symptomatology: Insights from Postmenopausal Women. Menopause.

[56] Huang W.-Y., Hsin I.-L., Chen D.-R., Chang C.-C., Kor C.-T., Chen T.-Y., Wu H.-M. (2017). Circulating Interleukin-8 and Tumor Necrosis Factor-α Are Associated with Hot Flashes in Healthy Postmenopausal Women. PLoS ONE.

[57] Deecher D.C., Dorries K. (2007). Understanding the Pathophysiology of Vasomotor Symptoms (Hot Flushes and Night Sweats) That Occur in Perimenopause, Menopause, and Postmenopause Life Stages. Arch. Womens Ment. Health.

[58] Harvie M.N., Pegington M., Mattson M.P., Frystyk J., Dillon B., Evans G., Cuzick J., Jebb S.A., Martin B., Cutler R.G. (2011). The Effects of Intermittent or Continuous Energy Restriction on Weight Loss and Metabolic Disease Risk Markers: A Randomized Trial in Young Overweight Women. Int. J. Obes..

[59] Li C., Xing C., Zhang J., Zhao H., Shi W., He B. (2021). Eight-Hour Time-Restricted Feeding Improves Endocrine and Metabolic Profiles in Women with Anovulatory Polycystic Ovary Syndrome. J. Transl. Med..

[60] Kalam F., Akasheh R.T., Cienfuegos S., Ankireddy A., Gabel K., Ezpeleta M., Lin S., Tamatam C.M., Reddy S.P., Spring B. (2023). Effect of Time-Restricted Eating on Sex Hormone Levels in Premenopausal and Postmenopausal Females. Obesity.

[61] Steenstrup B., Le Rumeur E., Moreau S., Cornu J.N. (2018). [Sedentary lifestyle and urinary incontinence in women: A literature review]. Prog. Urol..

[62] El-Bandrawy A. (2021). Effect of Aerobic Walking Exercise on Stress Urinary Incontinence in Postmenopausal Women. Women Sport Phys. Act. J..

[63] Lara L.A.d.S., Montenegro M.L., Franco M.M., Abreu D.C.C., Rosa e Silva A.C.J., Ferreira C.H.J. (2012). Is the Sexual Satisfaction of Postmenopausal Women Enhanced by Physical Exercise and Pelvic Floor Muscle Training?. J. Sex. Med..

[64] Soriano-Maldonado A., Díez-Fernández D.M., Esteban-Simón A., Rodríguez-Pérez M.A., Artés-Rodríguez E., Casimiro-Artés M.A., Moreno-Martos H., Toro-de-Federico A., Hachem-Salas N., Bartholdy C. (2023). Effects of a 12-Week Supervised Resistance Training Program, Combined with Home-Based Physical Activity, on Physical Fitness and Quality of Life in Female Breast Cancer Survivors: The EFICAN Randomized Controlled Trial. J. Cancer Surviv..

[65] Mathisen T.F., Rosenvinge J.H., Friborg O., Vrabel K., Bratland-Sanda S., Pettersen G., Sundgot-Borgen J. (2020). Is Physical Exercise and Dietary Therapy a Feasible Alternative to Cognitive Behavior Therapy in Treatment of Eating Disorders? A Randomized Controlled Trial of Two Group Therapies. Int. J. Eat. Disord..

